# Genomic analysis of fruit size and shape traits in apple: unveiling candidate genes through GWAS analysis

**DOI:** 10.1093/hr/uhad270

**Published:** 2023-12-19

**Authors:** Christian Dujak, Veredas Coleto-Alcudia, Maria José Aranzana

**Affiliations:** Centre for Research in Agricultural Genomics (CRAG) CSIC-IRTA-UABUB, Plant and Animal Genomics, Campus UAB, 08193 Bellaterra, Barcelona, Spain; Centre for Research in Agricultural Genomics (CRAG) CSIC-IRTA-UABUB, Plant and Animal Genomics, Campus UAB, 08193 Bellaterra, Barcelona, Spain; Centre for Research in Agricultural Genomics (CRAG) CSIC-IRTA-UABUB, Plant and Animal Genomics, Campus UAB, 08193 Bellaterra, Barcelona, Spain; IRTA (Institut de Recerca i Tecnologia Agroalimentàries), Genomics and Biotechnology, 08140 Caldes de Montbui, Barcelona, Spain

## Abstract

Genomic tools facilitate the efficient selection of improved genetic materials within a breeding program. Here, we focus on two apple fruit quality traits: shape and size. We utilized data from 11 fruit morphology parameters gathered across three years of harvest from 355 genotypes of the apple REFPOP collection, which serves as a representative sample of the genetic variability present in European-cultivated apples. The data were then employed for genome-wide association studies (GWAS) using the FarmCPU and the BLINK models. The analysis identified 59 SNPs associated with fruit size and shape traits (35 with FarmCPU and 45 with BLINK) responsible for 71 QTNs. These QTNs were distributed across all chromosomes except for chromosomes 10 and 15. Thirty-four QTNs, identified by 27 SNPs, were related for size traits, and 37 QTNs, identified by 26 SNPs, were related to shape attributes. The definition of the haploblocks containing the most relevant SNPs served to propose candidate genes, among them the genes of the ovate family protein MdOFP17 and MdOFP4 that were in a 9.7kb haploblock on Chromosome 11. RNA-seq data revealed low or null expression of these genes in the oblong cultivar “Skovfoged” and higher expression in the flat “Grand’mere.” The Gene Ontology enrichment analysis support a role of OFPs and hormones in shape regulation. In conclusion, this comprehensive GWAS analysis of the apple REFPOP collection has revealed promising genetic markers and candidate genes associated with apple fruit shape and size attributes, providing valuable insights that could enhance the efficiency of future breeding programs.

## Introduction

Domesticated apples belong to the diploid species *Malus x domestica* (Suckow) Borkh. with a haploid chromosome number *x* = 17 and a highly duplicated genome of 651 Mb [1]. Parentage analysis performed in a large collection of European apple genotypes revealed a dense pedigree network with few key varieties highly used as founders at the top of the European pedigree, which remounts to few generations back [2, 3]. Also, the contribution of the founders and their derived varieties to the overall pedigree was unequal.

Currently, a limited number of varieties dominate apple production and breeding, leading to a reduction in genetic diversity among commercial cultivars compared to that found in domesticated apples [4]. In breeding programs, fruit quality and productivity have traditionally been among the main objectives, while the recent need for varieties adapted to the effects of climate change (such as water scarcity, higher temperatures, and emerging diseases) demands more efficient and innovative breeding strategies, including novel phenotyping methods and molecular markers [5]. The use of molecular markers has enabled efficient selection in apple breeding, with different approaches being adopted in commercial breeding programs [6]. However, cutting-edge scientific development and the availability of materials and genomic tools are essential for the progress of these breeding programs.

An essential tool for apple breeding is the apple REFPOP, a European collection of 534 genotypes (accessions and progenies) that represent the current European breeding germplasm. This collection was genotyped with high-density SNP arrays and evaluated over years in six European countries to study the environmental effect on the genotypes [7]. Using the apple REFPOP phenotypic and genotypic data, Jung *et al.* [8] conducted genome-wide association (GWAS) and genomic selection (GS) studies, identifying important QTNs associated with numerous traits, including flowering time; harvested date; productivity; and fruit traits such as color, russeting, bitter pit, and fruit size, which need to be validated for use in breeding.

In addition to the above study, several works have aimed at identifying DNA polymorphisms associated with apple traits. Chagné *et al.* [9] compiled a list of 128 single-nucleotide polymorphisms (SNPs) for validation in a panel of accessions, including commercial varieties, advanced selections, and seedlings. Some of the SNPs were highly associated with relevant traits, making them suitable for molecular breeding.

Most of the works have been addressed to identify markers associated to disease resistance genes and to fruit quality traits like color, acidity, firmness, or compounds related to flavor in apple. However, few publications have focused on fruit size and shape. Globally traded apples must follow specific criteria, including diameter and uniformity, with deviations leading to lower-quality classifications. While breeders consider diverse shapes, adhering to standard descriptors is crucial for cultivar distinctiveness and stability assessments, emphasizing the importance of genomic research. The identification of genes or genomic regions that regulate these features will facilitates the precise selection of desired apple varieties through marker-assisted selection (MAS), ensuring compliance with market-quality standards.

Significant progress has been made in understanding the genetic inheritance and regulation of fruit shape in vegetable crops. For instance, studies have identified several genes and QTLs that control the ovary and fruit elongation in tomato, such as SUN, OVATE, and FS8.1 [10–12]. However, advances in apple fruit size and shape traits have been limited to the identification of a few molecular markers (SSRs and SNPs) located along the apple genome, except for Chromosome 6 [13–19]. Such markers were primary found in bi-parental families and their efficiency for MAS needs to be validated. In addition, only few QTLs for fruit shape, measured as the ration between width and heigh (i.e. fruit shape index, FSI), have been identified in segregating populations [20–22]. Besides FSI, other shape features, such as fruit shape triangle (FST) representing conicity, as well as the angles at the eye and calix (distal and proximal angle macro, DAM and PAM), and the roundness (eccentricity, ECC), have been identified as shape component descriptors [23]. However, till now, they have not been considered in genomic studies.

To enhance our understanding of the genomic regions, markers, and genes responsible for the inherited natural variation of fruit morphology, we conducted a GWAS study using fruit measurements obtained for a comprehensive description of fruit shape and size by Dujak *et al*. [23] in the densely genotyped apple REFPOP collection [7,8]. Also, whole-genome RNA-seq data served to propose candidate genes that will require further validation.

## Results

### Phenotypic data

The traits evaluated are broadly described in Dujak *et al.* [23]. For each trait, density plots were created to visualize how the data were distributed in each specific year. In addition, a density plot was generated to represent the mean distribution per trait across all years and give a sense of the overall trend or central tendency when combining the 3-year data (Supplementary Fig. S1). Trait distributions tended to exhibit similarities in terms of their central tendencies and spread, suggesting that the traits being evaluated did not show significant changes or variations across the years and that mean values represented well the overall trend.

**Figure 1:**
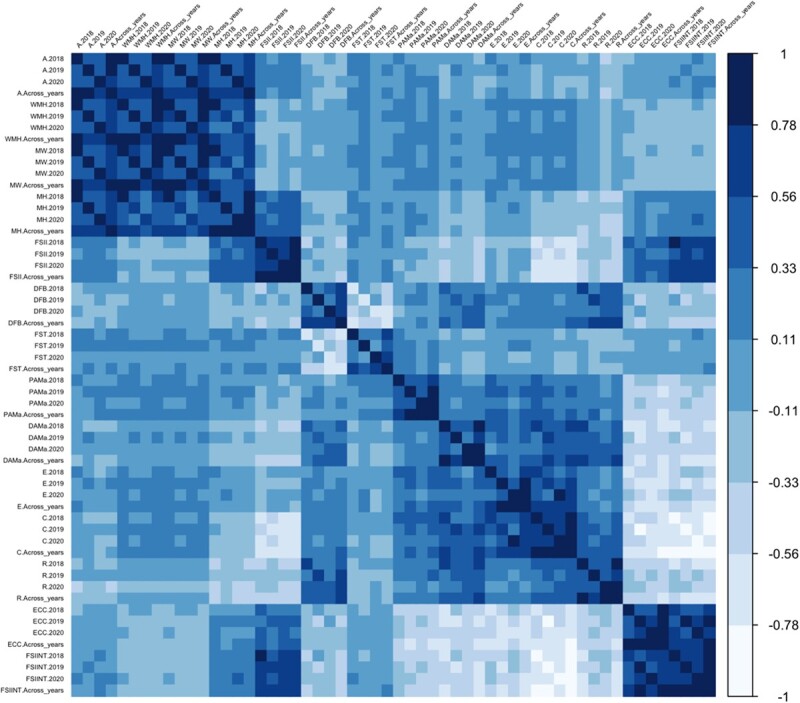
Spearman correlation analysis of fruit size and shape traits across multiple years, as well as their mean values across all years. The traits evaluated area (A), width mid-height (WMH), maximum width (MW), maximum height (MH) fruit shape index external I (FSII), distal fruit blockiness (DFB), fruit shape triangle (FST), proximal angle macro (PAMa), distal angle macro (DAMa), ellipsoid (E), circular (C), rectangular (R), eccentricity (ECC), and fruit shape internal (FSIINT). See correlation coefficients in Supplementary Fig. S2.

A comprehensive correlation analysis between traits, years, and the mean across years, revealed correlations ranging from moderate to strong (Fig. 1, Supplementary Fig. S2). When considering the correlations between year and the mean across years values for a given attribute, the lowest value was found for the FST observations in 2019 (*r* = 0.51) while the highest correlation was observed for FSII in 2020 (*r* = 0.91) (Supplementary Fig. S2).

### Genome-wide association studies

**Figure 2:**
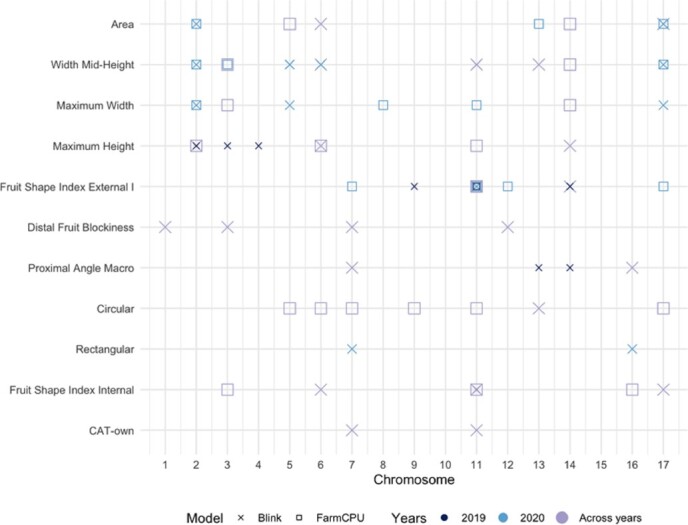
Summary of GWAS results for the size and shape traits using different models. QTNs obtained with Blink (x) and FarmCPU (□) models in the datasets for 2019 (●), 2020 (●), and mean values (●) are represented. The X-axis corresponds to the chromosomes, while the Y-axis represents the traits.

GWASs were conducted for all traits using the per year as well as the mean across-years values using two models (FarmCPU and BLINK) (Fig. 2). The results are displayed in Manhattan and QQ plots in the Supplementary Figs S3 and S4, respectively. The GWAS analysis identified SNPs with association values surpassing the Bonferroni threshold (−log10(*p*) = 6.751) for all traits except for the fruit shape triangle (FST), for the distal angle macro (DAMa), ellipsoid (E), and for the eccentricity (ECC). Considering the two GWAS models, the 3 years of data and the mean across-years values, we identified 59 SNPs associated (35 with FarmCPU and 45 with BLINK) responsible for 71 QTNs (Fig. 2, Supplementary Table S1). Thirty-nine of the QTNs (55%) were identified when using the mean values. Five QTNs were identified simultaneously with two datasets (in all cases were QTNs detected with the 2020 and with the mean values datasets), and nine QTNs were identified by the two models in either one of the year’s assessments (six QTNs) or when using the means (three QTNs).

In total, seven SNPs were simultaneously associated with more than one attribute, being one of the SNPs associated with three (AX-115482211 on Chromosome 2:21759081; with A, MW, and MWH). The 71 QTNs were distributed along all but the 10 and 15 chromosomes, ranging from 2 to 13 per chromosome. While some QTNs were scattered along the chromosome, others were in clusters.

### QTNs for size-related traits

Overall, our analysis revealed 34 QTNs significantly associated with size-related traits (Fig. 2 and Supplementary Table S1). Among these, 12 QTNs were linked to width mid-height (WMH), eight to maximum height (MH), seven to maximum width (MW), and seven to area (A). A majority of the QTNs were discovered either in the dataset for the year 2020 or in the mean data encompassing all years. These QTNs were characterized by 27 SNPs, with five of these SNPs being associated with multiple QTNs. This is the case of the SNPs AX-115482211, on Chromosome 2:21759081, and AX-115481999 on Chromosome 3:30093454, identifying three QTNs each, and the SNPs AX-115378078 on Chromosome 6:35186920, AX-115295642 on Chromosome 14:23185565, and AX-115312607 on Chromosome 17:2796935, identifying two QTNs each.

Among the discovered SNPs, four exhibited simultaneous significance for both MW and WMH, localized on chromosomes 2, 3, 14, and 17. The SNP on Chromosome 2 (AX-115482211) also demonstrated significance for the Area (A) trait (Supplementary Table S1). Moreover, the QTNs linked to WMH were distributed across eight different chromosomes.

### QTNs for shape-related traits

The study revealed 37 QTNs associated with shape-related traits, distributed across 12 chromosomes. Specifically, we identified 12 QTNs linked to the fruit shape index external I (FSII), 7 associated with the circular measure (C), 6 with the fruit shape index internal (FSIINT), and 4 QTNs for each of the proximal angle macro and distal fruit blockiness measures. Additionally, we detected two QTNs each for the CAT-own and rectangular values.

Two significant SNPs on Chromosome 11 located ~32 Mb apart (AX-115335214 and AX-105213957; in positions 4947462 and 37649389, respectively) and one SNP on Chromosome 14:291347 (AX-115336086) was responsible for six FSII QTNs for either the 2020 or the mean data. Furthermore, Chromosome 11 contained nine QTNs associated with C, FSII, FSIINT, and CAT-own spaced along the chromosome; four QTNs (one for FSIINT, one for CAT-own, and two for FSII) were in a region of 248 kb (Supplementary Table S1).

Twenty-three of the QTNs were found when using the mean values and 14 QTNs with the 2019 and 2020 datasets. Four out of the thirty-two SNPs were found simultaneously with the 2020 and the mean values datasets.

### Phenotypic variation and haploblocks

**Figure 3:**
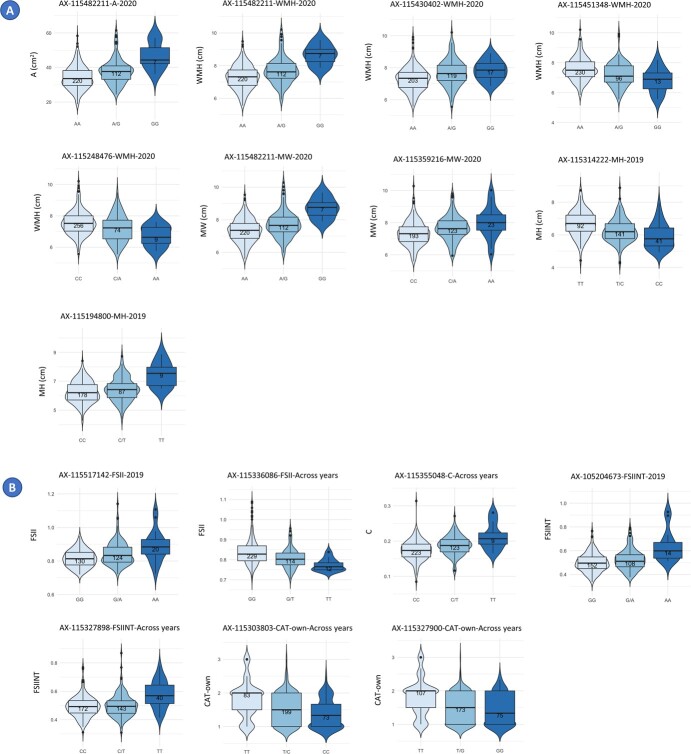
Violin plots displaying the frequency distribution of size (**A**) and shape (**B**) phenotypic values across genotypes. Each violin plot corresponds to a specific SNP-QTN combination, illustrating the distribution of trait values for different genotypes. In each violin plot, the X-axis represents the genotypes, with the first allele (on the left) indicating the homozygous genotype for the reference allele in the GDDH13 whole genome v1.1, the middle representing the heterozygous genotype, and the right side representing the homozygous genotype for the alternative or minor allele.

The phenotypic variation explained (PVE) by individual SNP spanned from 0.03% to 12.51%, with a mean PVE of 3.74% (Supplementary Table S2). To visualize the genotype–phenotype relationship for each SNP and QTN, we used violin plots. Figure 3 illustrates 14 violin plots, each capturing the phenotypic differences among the three genotypic classes: homozygous for the reference allele, heterozygous, and homozygous for the alternative allele.

The SNP AX-115482211 displayed concurrent associations with three fruit size measures: area, width-mid height, and maximum width. Individuals carrying the alternative allele G (with an allele frequency of 19%) exhibited significantly larger fruits compared to those with the reference allele. This effect was evident in both heterozygous and homozygous individuals (Fig. 3A and Supplementary Table S3).

To investigate the top 10 most outstanding SNP-QTN combinations, we conducted haplotype analysis by constructing haploblocks centered around the SNP. Due to linkage between two of the SNPs, we obtained a total of nine distinct haploblocks. These haploblocks were distributed in six chromosomes (2, 4, 6, 7, 11, and 13) and exhibited an average size of 31.5 kb, with lengths ranging from 1.1 to 111 kb. In total, 13 QTNs were found within these haploblocks (Supplementary Table S4).

**Figure 4:**
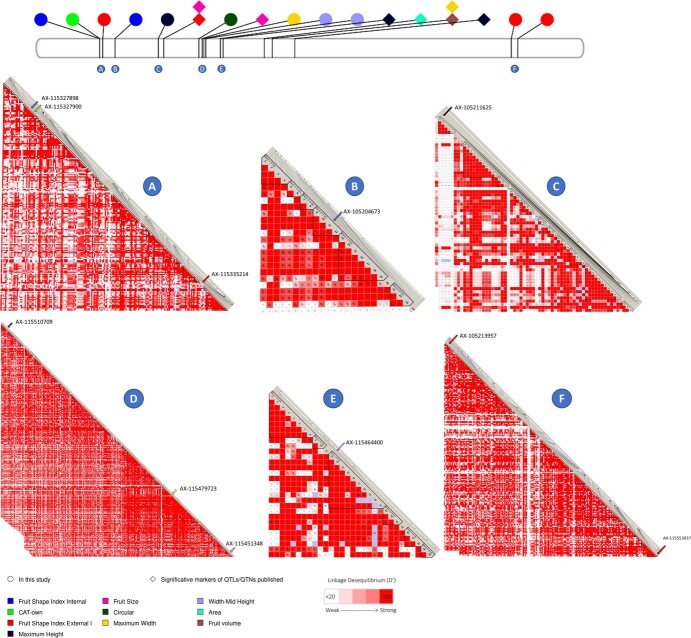
Linkage disequilibrium along Chromosome 11. The top figure displays Chromosome 11 from GDDH13v1.1, with position of markers published in this study and symbols representing QTNs found in this study (circles) and published (kites). The colors of the circles correspond to the associated QTN, and each circle is labeled with a letter representing the respective haploblock. Haploblocks, defined using GDDH13v1.1 positions, are as follows: Haploblock A: 4.661.534 to 4.958.286 (199 markers), Haploblock B: 5.930.883 to 5.948.789 pb (24 markers), Haploblock C: 9.046.670 to 9.556.662 pb (63 markers), Haploblock D: 12.638.696 to 13.198.304 pb (434 markers), Haploblock E: 14.355.224 to 14.402.233 pb (30 markers), and Haploblock F: 37.648.782 to 38.174.120 bp (241 markers). The color gradient from white to red represents the level of linkage disequilibrium (D′). A D′ value <20 is considered weak linkage disequilibrium, and red color indicates a D′ value of 100, representing strong linkage disequilibrium.

A notable cluster of QTNs for both size and shape attributes occurred within a genomic region spanning 1.9 Mb along Chromosome 11. Among these QTNs, 11 were found within haploblocks, linked or co-segregating (Fig. 4).

**Figure 5:**
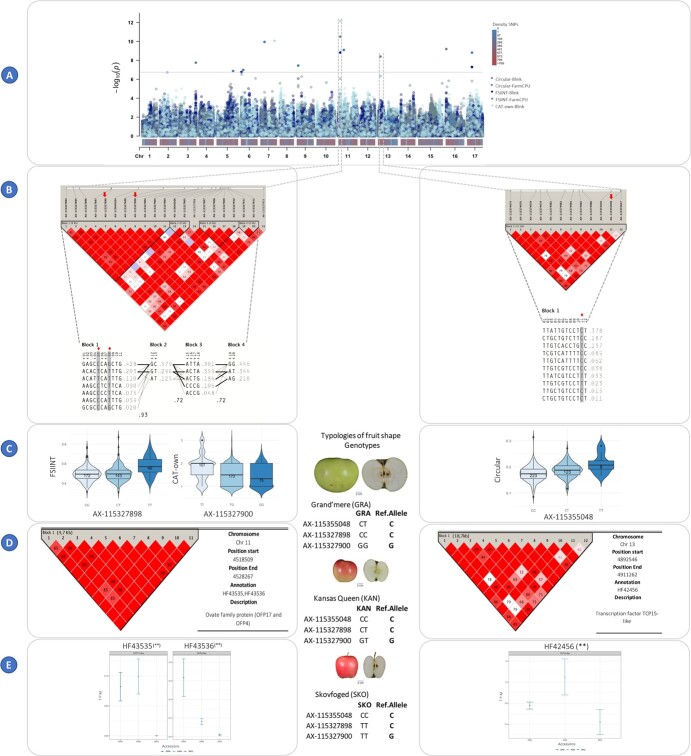
Integrated analysis of GWAS results, haploblocks, genotype–phenotype frequency, gene annotation, and RNA-seq, for FSIINT, CAT-own and Circular traits with the mean across years data. (**A**) GWAS results: this panel showcases multiple Manhattan plots representing the GWAS results for the three traits: FSIINT, CAT-own and circular. The plots depict the significance of genetic markers on each chromosome, colored based on the trait and the two models used (Blink and FarmCPU). Density plots illustrate SNP distribution on each chromosome. (**B**) Haploblocks & Haplotypes: the linkage disequilibrium (D′) based on the GDDH13v1 genome is presented. Haploblocks are identified using the criteria from Gabriel *et al.* [56], with colors indicating the strength of D′ (white = weak, red = strong). The haplotypes of each block and their allelic frequency are shown below. (**C**) Frequency Genotype–Phenotype: Allele frequency for the three traits is displayed, along with the corresponding apple shape genotypes (“Grand’mere” = flat, “Kansas Queen” = round, “Skovfoged” = oblong). (**D**) Gene annotation: This section presents the candidate genes annotated within the haploblock, utilizing the annotations from the HFTH1 whole genome v1.0. (**E**) RNA-seq: TPM data at the 13 DAA fruit stage for three candidate genes (HF43535, HF43536, and HF42456) across the three genotypes.

In Fig. 5A, we present three QTNs related to fruit shape, highlighting the respective haploblocks and their haplotype frequencies for three of the most significant associated SNPs: AX-115327898 (C/T alleles) and AX-115327900 (G/T alleles), both highly linked, situated 5 kb apart at the top of Chromosome 11 (positions 4699021 and 4703926) and associated to FSIINT and CAT-own attributes, respectively. Additionally, we identified AX-115355048 on Chromosome 13:5186503, associated with the circular measure provided by the Tomato Analyzer software, which describes the extent to which the fruit section resembles a circle (Fig. 5B).

Apples from cultivars with the allele T in AX-115327898 in homozygosis exhibited significantly higher FSIINT values, indicating a clear tendency for oblong fruit shapes. By contrary, individuals with CC and CT genotypes at this site produced flat and circular fruits (accessions such as “Grand’mere” and “Kansas Queen”). Similarly, apples from cultivars homozygous for T in the SNP AX-115327900 such as “Skovfoged” showed oblong shapes, while apples of heterozygous GT or homozygous GG cultivars were predominantly flat (as “Grand’mere”) or circular and “Kansas Queen”) (Fig. 5C). These two SNPs were in complete LD (D′ = 1) and occurred in a haploblock 9.7-kb long, which had seven haplotypes with an average frequency of 0.14, ranging from 0.02 to 0.428 (Fig. 5B).

The haploblock containing the SNP AX-115355048 on Chromosome 13 (with CT alleles) was significantly associated with the circular attribute. The haploblock was 18.7-kb long and included 10 haplotypes with frequencies ranging from 0.378 to 0.011. Cultivars homozygous CC showed lower circular values and higher FSIINT (see Fig. 5C).

### Gene annotation

For each of the 59 associated SNPs, we searched for annotated genes within a 200-kb region (100 kb upstream and downstream the SNP position) in the HFTH1 whole genome v1.0. This gene annotation analysis revealed 873 annotated genes, with 371 genes linked to size QTNs and 502 to genes linked to shape QTNs. Additionally, 53% of the annotations contained a molecular description, according to Gene Ontology (GO) databases. Fifty-one genes had protein-binding molecular function; 40 genes were related to biological processes, including transcriptional regulation, DNA repair, phosphorylation, and transmembrane transport, among others. Moreover, we found genes that play vital roles in cell division; growth; cell modification; and response to hormones, such as gibberellin, auxin, and ethylene.

Based on the TAIR database, a subset of genes was found to be directly linked to fruit development and growth. Specifically, we identified nine genes related to auxin response, including HF06172, HF40493, HF29276, HF02793, HF08237, HF41541, HF02644, HF02646, and HF12008. Four genes were related to ethylene response (HF14170, HF14173, HF16534, HF11991); three genes were involved in the gibberellins regulatory network (HF41950, HF38795, HF08230). Additionally, we recognized two genes related to fruit shape, including the Ovate Family protein HF43535 and HF43536 (Supplementary Table S5).

Gene annotation in the nine haploblocks previously defined based on linkage disequilibrium identified a total of 30 genes according to the TAIR database. Notably, we found among these the Ovate Family Proteins 17 (OFP17) and 4 (OFP14) (HF43535, HF43536), the TCP15-like transcription factor involved in plant regulation (HF42456), and several proteins of the kinase superfamily (Fig. 5D, Supplementary Tables S4 and S5).

### Haploblock differential gene expression analysis

Whole RNA sequence data of three genotypes, one oblate (“Grand’mere”), one round (“Kansas Queen”) and one oblong (“Skovfoged”) obtained from fruits at 13 days after anthesis were analyzed to evaluate the expression in fruit of the 30 genes annotated in the haploblocks (Supplementary Table S5). Twenty-three of them were transcriptionally expressed in fruits of the three genotypes. A total of six genes exhibited differential expression between the genotypes: the genes HF43535 and HF43536 (OFP17 and OFP14, respectively) were annotated within the haploblocks of the SNPs AX-115327898 and AX-115327900 on Chromosome 11. The genes HF10079 and HF10080 (belonging to the Patched family and protein kinase proteins, respectively) were found in the haploblocks of the SNPs AX-115513701 and AX-115448691 SNPs on Chromosome 6. The gene HF15994 (encoding an unknown function protein) was identified in the haploblock of the SNP AX-115194800 on Chromosome 4, while the gene HF42456 (a transcription factor TCP15-like) was in the haploblock of the SNP AX-115355048 on Chromosome 13.

Among these genes, three have been described to play a crucial function in organ regulation and development: the OFP17, the OFP4, and the *TCP15-like gene*. For the OFP17, significant differences in expression were observed between the “Grand’mere” (flat) and “Skovfoged” (oblong) (GRAvsSKO) and between “Kansas Queen” (round) and “Skovfoged” (KANvsSKO). However, no significant differences in expression were found between “Grand’mere” and “Kansas Queen” (GRAvsKAN). This gene was expressed at a lower level in the oblong variety “Skovfoged” (Fig. 5E and Supplementary Table S6). The HF43536 gene (OFP4) was differentially expressed in the pairs GRAvsKAN (oblate and round) and GRAvsSKO (oblate and oblong), with higher RNA levels in the flat genotype.

As a mean to validate the RNA-seq data, the gene expression of this last gene (HF43536) was assessed by RT-qPCR, obtaining an Eff = 2 and an *r*-squared of 0.8591 between the cycle threshold (Ct) and log2 [transcript per million (TPM)] values (Supplementary Fig. S5).

Transcription factor TCP15-like gene (HF42456) showed differences in gene expression levels between the oblate and oblong fruits (GRAvsSKO) and between round and oblong fruits (KANvsSKO), with the SKO genotype showing lower gene expression (Fig. 5E and Supplementary Table S6).

### Whole-genome differential gene expression analysis

When expanding the differential gene expression analysis to total RNAseq data, we found 52 differentially expressed genes (DEGs) between “Grand’mere” and “Kansas Queen”, 2761 between “Grand’mere” and “Skovfoged,” and 2316 between “Kansas Queen” and “Skovfoged” (Supplementary Fig. S7A–D). Meanwhile, the GO enrichment analysis (GOEA) yielded a limited number of GO terms between “Grand’mere” and “Kansas Queen” (Supplementary Fig. S7E, an enrichment emerged in GO and KEGG terms related to the regulation DNA-templated transcription and plant hormone metabolism for the other two pairs of comparison (Supplementary Fig. S7F and G).

A hierarchical clustering analysis (HCA) of gene expression profiles for DEGs revealed distinct expression patterns among different fruit shapes, leading to the identification of nine major clusters of expression profiles (Supplementary Fig. S8A, Supplementary Table S8). Focusing on our primary candidates for fruit shape regulation, both MdOFP4 (HF43536) and MdOFP17 (HF43535) were identified in cluster 8, which comprises downregulated genes in oblong apples. Cluster 8 also included other genes related to shape regulation, such as microtubule-associated proteins, IQ67 family proteins, cyclins, and genes associated with gibberellin and brassinosteroid synthesis. The GOEA of this cluster revealed terms related to microtubule and cytoskeleton organization, responses to auxins and cell wall organization (Supplementary Fig. S8C). Opposite expression pattern profiles were grouped in 7 and 9 clusters (Supplementary Fig. S8B and D), where GO terms related to hormone metabolism and signaling and regulation of DNA-templated transcription were also revealed.

## Discussion

Fruit shape, in particular the shape of the apple, is relevant both for the description and varietal characterization as well as for aspects related to its commercialization and market value. Although visual and “easy-to-evaluate” criteria as the FSII for fruit classification are useful for the above-mentioned purposes, more objective data and precise phenotyping are necessary to sufficiently characterize loci and the underlying genes that contribute to shape variation [24] perform genome studies. Here, we used the data and measures obtained and described in Dujak *et al*. [23] to search for genomic regions controlling apple fruit shape and size attributes.

Fruit size and shape data were obtained in thousands of images acquired in fruits of a total of 355 genotypes in three consecutive harvesting campaigns (93 genotypes were common in the 3 years of assessments) [23]. Several of the evaluated traits showed high heritability values, indicating a substantial influence of genetic factors on fruit morphology. It is broadly accepted that climatic and management factors affect fruit shape and size, although some studies show low differences in the FSII ratio between years, only observed under high divergences in the air and soil temperature in spring, for it may cause differences in the fruit seed number, the main factor determining fruit shape [25]. In the Spanish apple REFPOP location, spring temperatures were only moderately milder in spring 2020, compared to 2018 and 2019, so we shouldn’t expect extreme divergences. The correlations between the values obtained each year support this fact.

GWAS analysis yielded significant associations for most of the studied traits, but there were a few exceptions where no significant associations were found. This finding might suggest that these traits have weaker genetic associations or that other factors beyond the examined SNPs play a more dominant role in influencing their expression or variation.

**Figure 6:**
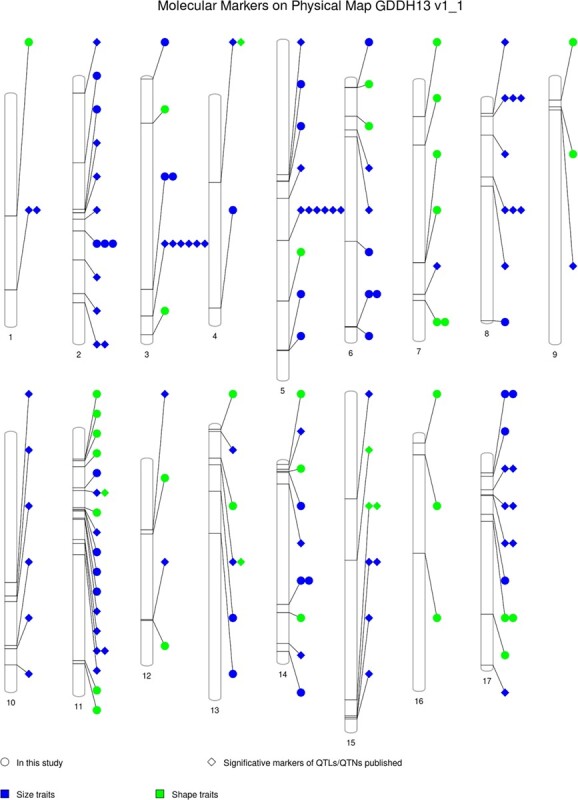
PhenoGram of the molecular markers on the physical map according to the apple GDDH13 whole genome v1.1. Significant markers mapped for apple fruit measures, including markers published in QTLs/QTNs analysis and the QTNs found in this study. Symbols: circle corresponds to “in this study” and kite, “significative markers of QTLs/QTNs published.” Color blue for size traits and green for shape traits. See details in Supplementary Figure S6.

In our study, we made significant discoveries of several SNPs associated with size- and shape-related traits. Joining these markers with markers and QTLs published in other studies [8, 13, 16, 21, 22], we have constructed a PhenoGram with 110 molecular markers (SNPs and SSRs) (Fig. 6, Supplementary Table S7), 76 for size and 37 for shape traits.

### Size-associated markers

Several size-associated markers discovered here mapped close to other previously published. For example, on Chromosome 3, Potts *et al.* [16] identified two QTLs responsible for fruit circumference and height in a segregating family. These QTLs collectively explained 45% of the phenotypic variation observed. Here, we identified two QTNs (for MS and WMH) at 3.8-Mb distance, with the gene HF40493 in the vicinity. This gene is a notable auxin response factor, sharing homology with *AtARF4* (identified by Liu *et al.* [26]), that plays a crucial role in regulating both female and male gametophyte development in Arabidopsis, as evidenced by the research conducted by Bu *et al.* [27].

Also, on Chromosome 5, we found eight QTNs. Two of them (one for A and one for WMH) were at a very close distance (82 kb apart). The two associated SNPs (AX-115248476 and AX-115435503) were in LD (R-squared mean was 0.38) and added up to 10.6% of PVE. These QTNs were at ~15.7 Mb from a QTL for fruit maximum heigh reported by Potts *et al.* [16] with an LOD of 3.94 and 21.5% of the variance explained. Two other QTNs for fruit width were at the top of this chromosome, with the two SNPs (AX-115638603 and AX-115436710) 102 kb apart and at less than 1 Mb from a QTL for the same attribute identified by Chang *et al.* [21] with an LOD of 2.9 explaining 9.2% of the variance, and 2.3 Mb apart from a QTL also for fruit width identified by Kenis *et al.* [13] with an LOD of 3.5 and 12.4% of the variance explained. The SNPs explained together 6.57% of the variance. Similarly, Potts *et al.* [16] identified a width QTL 8.4 Mb downstream of these SNPs.

Some genes annotated in these regions are responsible for growth regulation such as transcriptional factor B3 family protein/auxin-responsive factor AUX/IAA-related (HF12008), ethylene-responsive element binding factor 1 (HF11991), gibberellin-regulated family protein (HF08230), and auxin-responsive GH3 family protein (HF08237). Hormones, play an important role in fruit growth and are controlled by multiple genes. For example, endogenous auxin concentration is one of the factors controlling fruit size in apple [28]. In agreement with this, Devoghalaere *et al.* [14] suggest a potential role in fruit size of the Auxine Responsible Factor (ARF106) gene, contained in a QTL for fruit weight on Chromosome 15.

Chromosome 11 contains the highest number of markers (19) in the PhenoGram. For example, one of the associated SNPs here with width mid-height (AX-115464400) is only 216 kb apart from the SNP AX-115380060 associated with fruit size in Jung *et al.* [8]. Also, the confidence interval of a fruit size–related QTL on this chromosome contains the miRNA172. The overexpression of this miRNA has a negative effect in fruit size [29].

Additional QTLs/QTNs for fruit size attributes have also been reported along chromosomes 2, 8, 13, 14, and 17 in this study as well as in [8, 13, 14, 21].

Also in the apple REFPOP, Jung *et al.* [8] identified 15 SNPs linked to two of the size parameters studied here (fruit diameter and length). Although in some cases, they were close to the SNPs identified here, they were not coincident. In our research, we use a subsample of the apple REFPOP. Given the inherent genetic diversity within this collection, there exists the potential for allele frequency changes in certain SNPs, which could, in turn, impact the statistical significance of the associations. To mitigate this variability and enhance the robustness of our analysis, we undertook the task of calculating haplotypes surrounding the SNPs under investigation. Haplotype-based methods, by considering combinations of SNPs within haplotypes, are practical solutions for MAS when diagnostic markers are unavailable or are not informative enough [24].

### Shape-associated markers

In this study, several QTNs for apple fruit shape attributes, measured through the analysis of bidimensional images, have been identified. Among the most relevant associations, we found a 9.7-kb haploblock on Chromosome 11 with QTNs for FSIINT and CAT-own traits. The two associated SNPs (AX-115327898 and AX11532790) were in complete LD; the reference allele in homozygosis was present in the flat cultivars, while the alternative in homozygosis was preferentially observed in oblong fruits. Cao *et al.* [22] reported a QTL for the same measure (FSII), at 5-Mb distance from SNPs (AX-115327898 and AX-115327900) associated with FSII and another measure (CAT-own) that are highly correlated. Chang *et al.* [21] also detected several QTLs for the fruit shape index (FSI); one of those QTLs in LG11 contributed to a phenotypic variance between 10.3% and 13.7% in a segregating population. This haploblock contained two ovate family protein genes (MdOFP17 and MdOFP4).

More than 25% of the QTNs identified here are related to the FSI (FSII and FSIINT studied here). This index has been preferentially used in successive works to describe fruit shape, since it is the measure with higher weight in the definition of fruit shape [23]. However, other attributes are good descriptors of the apple shape, as is the fruit circularity (C). For this trait, the most relevant QTN was identified within an 18.7-kb haploblock on Chromosome 13. The alternative allele of the associate SNP (AX-115355048) was preferentially observed in varieties bearing fruits with tendency to the oblong shape. This haploblock contained only one annotated gene, the TCP15-like transcription factor. This transcription factor is involved in the regulation of plant development and the stimulation of biosynthesis of hormones such as brassinosteroids, jasmonic acid, and flavonoids [30].

This study revealed the most relevant marker associations with the parameter FSI and the categorical classification CAT-own, both of which are highly correlated and widely recognize as fundamental descriptors of fruit shape. In addition to these primary descriptors, our investigation identified SNPs associated with other parameters, each contributing to the overall perception of fruit shape [23]. While these individual associations might not hold considerable utility for breeders in isolation, their combination may provide a more realistic prediction of the fruit when used in MAS. In this line, a study by Jurado-Ruiz *et al*. [31] used the SNPs identified in our research, in conjunction with additional previously published, to predict apple images using artificial intelligence models. This approach underscores the potential of combining a diverse array of SNP data for advanced and precise fruit shape prediction and selection in breeding programs.

### Candidate genes

The RNA-seq analysis of three varieties, each exhibiting contrasting phenotypes, revealed significant differences in their expression levels. For the gene MdOFP17, the oblong cultivar “Skovfoged” showed lower expression levels, while the flat variety “Grand’mere” showed an increase in the expression of MdOFP4. The two MdOFP genes occurred in cluster in the same haploblock.

The ovate family proteins are genes involved in the regulation of plant development in different organs, and in particular in the regulation of fleshy fruit shape, as described in several species such as tomato [32–34], pepper [35], melon [36], and peach [37]. They are transcriptional repressor genes, but they also play an important role in the regulation of cell division in tomato fruit development or in response to hormone changes [11, 38]. In Arabidopsis, tomato, and rice, the overexpression of OFPs causes the cotyledon, fruit, and seed to be flattened or, if there is a mutation in these genes, the organs are elongated [39].

In apples, the diversity of OFP genes (26) distributed in 13 chromosomes has been studied [40], but their role in apple fruit shape has not been described yet. Here, we provide some hints to understand how OFPs regulate the shape of apples through a transcriptomic point of view. The GOEA revealed an enrichment of regulation of DNA-template transcription and plant hormone metabolism terms in the comparisons including the elongated fruit variety “Skovfoged.” OFPs are specific players of the regulation DNA-templated transcription process, which supports a role of both, OFPs and hormones, in shape regulation.

Differential expression analysis and hierarchical clustering revealed the upregulation of MdOFP4 and MdOFP17 in flat apples, as well as genes from the IQ67 family protein, where SUN [10] belongs, that are related to microtubule organization, and the downregulation of genes related to the synthesis and signaling of hormones such as brassinosteroids and gibberellins, which have been linked to shape regulation in other species [35, 39, 41–45].

Further investigation into the functions and regulatory networks of these genes will shed light on their contributions to the observed differences in the studied plant populations.

## Conclusion

To date, few studies have been carried out to know which candidate regions or genes are responsible for fruit size and shape. In this study, we present QTNs and candidate genes for a better understanding of the genetic and molecular bases of apple fruit size and shape determination and highlight candidate genes, such as the MdOFP17 and MdOCP4, that may underlie the distinct fruit shapes observed among apple varieties. In addition, we provide here molecular markers for breeding.

## Materials and methods

### Plant materials

We used genotypic and phenotypic data of 355 genotypes of the apple REFPOP copy growing in Gimenells (Lleida, Spain), including 257 accessions and 98 seedlings derived from 31 families (Supplementary Information S1).

### Genotypic data

Genotypic data were extracted from Jung *et al.* [8] and consisted of 303 239 biallelic SNPs obtained with the Affymetrix Axiom® Apple 480 K SNP genotyping array [46], or imputed from the Illumina Infinium® 20 K SNP genotyping array [47] in accessions and progenies, respectively.

### Phenotypic data

Phenotypic data were extracted from Dujak *et al*. [23], and consisted on four size and 10 morphometric descriptors obtained using the Tomato Analyzer software Version 3 developed by Gonzalo *et al.* [48] (Supplementary information S2). The data were collected in 12 692 apple sections harvested over three seasons: 2018 (134 genotypes), 2019 (274 genotypes), and 2020 (339 genotypes). Of these, 94 genotypes were evaluated in all 3 years. The descriptors included measures of size, FSIs, fruit blockiness, fruit homogeneity, distal fruit end shape, and internal fruit eccentricity. We also used the CAT-own fruit classification system to assign fruits into oblate or flat (class value = 1), spheroid or round (class value = 2), and oblong classes (class value = 3) based on visual comparison with images of three standard fruit typologies.

At least three apples per clone (two clones per genotype) and year were evaluated to obtain raw data. Mean values for each genotype were used for the analyses. For genotypes evaluated in more than one harvest season, mean values were calculated for each measure, resulting in a final dataset of 355 genotypes (referred as mean across-years dataset) (Supplementary Data S1).

Spearman’s correlation for all datasets, the distribution of the data and the density plots, and heatmaps were calculated and plotted with the *ggplot2* package [49] in R Core Team (2022) program.

### Genome-wide association studies

GWAS**s** were conducted using two methods. The Fixed and random model Circulating Probability Unification method (FarmCPU) [50] and the Bayesian-information and Linkage-disequilibrium Iteratively Nested Keyway method (BLINK) [51]. FarmCPU combines the mixed linear model with the fixed-effect model (FEM) to control for confounding factors, such as kinship, and to reduce false negatives. It also incorporates the random effect model (REM) to select associated markers by maximum likelihood method, thus avoiding the over-fitting. BLINK, on the other hand, replaces REM with FEM and uses the Bayesian information criteria (BIC) based on the linkage disequilibrium to generate fewer false positives and high statistical power.

Both FarmCPU and BLINK were implemented in the R package GAPIT 3.0 [52]. GWAS was performed using genomic matrices with the same number of markers (303 239 SNPs) for four populations subsets with different sample sizes (as *n*_2018_ = 134 genotypes, *n*_2019_ = 274 genotypes, *n*_2020_ = 339 genotypes, and *n*_mean_ = 355). To control for population structure, we used three principal components. We filtered out SNPs with a minor allele frequency (MAF) < 0.05. To identify markers with significant association, we applied the Bonferroni correction with a significance threshold of *a* = α/𝑚, where α = 0.05 and *m* is the number of markers (−*log*10(*P*-value) > 6.75).

The resulting *P*-values were plotted in multiple Manhattan and QQ plots using the threshold described [53]. Significant QTNs for all datasets and methods were graphically represented along each chromosome using the ggplot2 package [49]. Additionally, the GAPIT output file provided the phenotype variance explained by SNP (PVE) and the MAF. To further investigate the relationship between genotype and phenotype, we calculated the coefficient of determination using a numerical coding of alleles (1 and 2 for homozygous alleles and 3 for heterozygous alleles). The allelic frequency of each significant SNP was calculated with its corresponding association (phenotype), represented in a boxplot using ggplot2.

### Haploblocks

We analyzed linkage disequilibrium (LD) and identified haploblocks using Haploview software [54] based on the position of significant SNPs that were filtered using PLINK [55]. We focused on a 200**-**kb window around the position of the SNP of interest (100 kb each side), using the GDDH13 v1.1 genome [1] as reference.

To identify haploblocks, we applied the following criteria: Hardy**–**Weinberg *P*-value cut-off, 0.01; minimum genotype cut-off, 0.75; maximum number of Mendel errors, 1; and minimum minor allele frequency, 0.05. We used the Gabriel *et al.* [56] criteria to determine the blocks, which require a minimum confidence interval for strong LD (D′) at the top of 0.95 and at the bottom of 0.2 (indicating the LD level from 0.2 to 1).

Using Haploview software**,** we calculated the allelic frequency of each haplotype in the population and identified connections between blocks.

### Candidate genes annotation

To annotate the genes in the haploblocks and the 200-kb regions flanking the associated SNPs (100 kb on both sides), we used the HFTH1 whole genome v1.0 [57] as the reference. For this, the haploblock regions initially aligned to the GDDH13 v1.1 whole genome assembly were subsequently aligned to the HFTH1 genome by BLAST+ from the GDR database [58]. To further annotate these genes, we utilized various databases, including GO terms [59], InterPro (IPR) [60], Kyoto Encyclopedia of genes and genomes (KEGG) orthologs and pathways [61], non-redundant proteins sequences from NCBI (RefSeq) [62], *Arabidopsis thaliana* orthologs from the Arabidopsis Information Resource (TAIR) [63], and the computer-annotated protein sequence database for the translation of coding sequences (UniProtKB/TrEMBL) [64].

Correlation between genes annotated in the HFTH1 and the GDH13 genomes can be found in Supplementary Table S9.

### RNA extraction and cDNA preparation

To investigate gene expression patterns, we collected apples from three genotypes with different shapes and sizes: “Grand’mere” (GRA), flat and large size; “Kansas Queen” (KAN), round and medium/small size; and “Skovfoged” (SKO), oblong and medium size. Three biological replicates of fruit samples were collected at 13 days after anthesis. All fruit samples were frozen in liquid nitrogen and stored at −80°C until further processing. Total RNA was extracted from the frozen samples using the Maxwell® RSC simplyRNA tissue kit and the Maxwell® RSC instrument and was purified twice with Turbo® DNase to remove any residual DNA contamination. The quality and quantity of the extracted RNA were assessed using the Bioanalyzer system, and the RNA was set to Novogene (London, England) for sequencing.

The RNA samples were converted to cDNA using the PrimeScript RT Reagent Takara kit. In the first step, the RNA was mixed with Oligo(dt) 20 nt (50 uM) and H_2_O RNase-free, and heated at 70°C for 5 min. In the second step, the cDNA synthesis reaction was performed using 5X PrimeScript Buffer, PrimeScript RT Enzyme, RNase out, dNTPs (100 uM), the first step reaction mixture, and H_2_O RNase-free and incubated at 50°C for 60 min followed by inactivation at 70°C for 15 min. The resulting cDNA samples were then verified for integrity and subsequent user by visualizing them on a 1.5% agarose gel and 1X TAE.

### Analysis of mRNA sequencing data

The mRNA sequencing libraries were subjected to quality control, with reads having a Phred score < 30 being removed. Illumina sequencing adapters were trimmed using Trim-Galore (https://www.bioinformatics.babraham.ac.uk/projects/trim_galore/) [65] version 0.6.1. Burrows–Wheeler Aligner [ 66] version 0.7.17 was used to map clean reads to the HFTH1 whole genome v1. High-quality RNA sequencing libraries were also mapped to HFTH1 by using HISAT [67] version 2.1.0. with default settings parameters. SAMStat [68] version 1.5.1 was used to analyze the quality of mapped and unmapped reads in Binary Alignment Map (BAM) files. The Mapping Quality Score (MAPQ) was used as an index to evaluate the quality of alignment and assembly, and only THE reads with MAPQ ≥ 30 were retained. SAMtools [69] version 1.9 was used to filter out multiple aligned reads and obtain statistic reports. Besides, filtered BAM files were transformed, indexed, and sorted according to the protocol needs using SAMtools. Quality control reports were generated before and after filtering and mapping with FastQC version 0.11.5 [70], and the results were summarized in an HTML file using MultiQC version 1.9 [71].

Gene quantification and count matrix construction were done with featureCounts [72] applying paired-end sequencing parameters. Chimeric count fragments were avoided, and the exon feature type was specified for read counting. The count matrix was annotated as transcript, and overlapping features were allowed for the differential use of exons during alternative splicing. The results were normalized to TPM.

To check for batch effects, the *sva* R package version 3.12 [73] was used. Preliminary exploratory analysis and visualization of the samples were also performed. The count matrix was normalized using a regularized logarithm transformation (*rlog*) to stabilize the variance across the mean for negative binomial data with a dispersion-mean trend and a low number of samples (*n* < 30).

The differential expression of genes (DEGs) annotated in the haploblock was analyzed using a Shapiro test to assess the normality of the distribution. For normally distributed data, an ANOVA-one way was applied, whereas a Kruskal–Wallis test was used for non-normal distribution. Differences between genotypes were determined by the Tukey HSD test, with a confidence level of *P* < 0.05, using the normalized count matrix in TPM.

### Total RNAseq data analysis

For differential expression analysis, samples were trimmed mean of M-values (TMM) normalized and statistical values calculated with the “EdgeR” package in R. DEGs were identified between varieties. Results were filtered for and adjusted *P*-value (FDR) < 0.05 and |logFC| > 2 in the pairwise comparisons. GOEA was performed with the “TopGO” and “GO.db” packages. KEGG pathway enrichment were performed with “clusterProfiler” package. Plots were drawn using “ggplot2” package of R. Hierarchical clustering analysis was performed with “tidyverse” package of R.

### Validation RNA-seq and expressed genes (transcript per million)

We validated the differential expression of the gene HF43536 using the primers Fw 5’-AGGGCAGCTAAGGATTTGGA-3′ and Rv 5’-TGTGTGTGCCATGTCAAACCAG-3. The qPCR was performed using the LightCycler 480 System Roche. Each reaction contained 5x MasterMix SYBR Green, primers Fw and Rv (each 10uM), H_2_0 nuclease-free and cDNA adjusted to dilution 1:40. The cycling conditions were pre-incubation at 95°C during 5 min, for amplification 40 cycles (at 95°C to 10 sec, 60°C to 10 sec**,** and 72°C to 30 sec), melting curve (at 95° to 5 sec, 65°C to 1 min) and finally cooling at 40°C to 1 min.

The amplification efficiency was calculated using the formula Eff = −1 + 10^(−1/slope), and the RNAseq data were validated by analyzing the R-squared between log2(TPM) and the cycle threshold (ct).

### PhenoGram

A phenoGram [74], based on chromosomal ideograms sharing the genomic information, was constructed using published SNPs or other molecular markers associated with apple shape and size. To locate them in the physical map the markers were first aligned using BLAST-NCBI [75] with the double haploid GDHH13 v1.1 reference genome. All QTNs obtained in this study were also included.

## Acknowledgements

C.D. was supported by “DON CARLOS ANTONIO LOPEZ” Abroad Postgraduate Scholarship Program, BECAL-Paraguay. V.C.A. is a recipient of grant PRE2019-088780 funded by MCIN/AEI/10.13039/501100011033 and by “ESF Investing in your future.” This research was supported by project PID2021-128885OB-I00 funded by MCIN/AEI/10.13039/501100011033 and by “ERDF A way of making Europe.” This project has received funding from the European Union’s Horizon 2020 research and innovation programme under grant agreement No 817970 (INVITE). We acknowledge support from the CERCA Programme (“Generalitat de Catalunya”), and the “Severo Ochoa Programme for Centres of Excellence in R&D” 2016-2019 (SEV-2015-0533) and 2020-2023 (CEX2019-000902-S) both funded by MCIN/AEI/10.13039/501100011033.

## Author contributions

M.J.A. and C.D. contributed to the design and implementation of the research and to the analysis of the results. C.D. conducted the laboratory experiments. V.C.A. performed the whole-genome RNAseq and the GCN analysis. All authors contributed to the writing of the manuscript.

## Data availability

Data are available in the Supplementary Data file.

## Conflict of interest statement

None declared.

## Supplementary data

Supplementary data are available at *Horticulture Research* online.

## Supplementary Material

Web_Material_uhad270
